# Prostate cancer castrate resistant progression usage of non-canonical androgen receptor signaling and ketone body fuel

**DOI:** 10.1038/s41388-021-02008-9

**Published:** 2021-09-28

**Authors:** Estefania Labanca, Juan Bizzotto, Pablo Sanchis, Nicolas Anselmino, Jun Yang, Peter D. A. Shepherd, Alejandra Paez, Valeria Antico-Arciuch, Sofia Lage-Vickers, Anh G. Hoang, Ximing Tang, Maria Gabriela Raso, Mark Titus, Eleni Efstathiou, Javier Cotignola, John Araujo, Christopher Logothetis, Elba Vazquez, Nora Navone, Geraldine Gueron

**Affiliations:** 1 Department of Genitourinary Medical Oncology and the David H. Koch Center for Applied Research of Genitourinary Cancers, The University of Texas MD Anderson Cancer Center, Houston, TX 77030 USA; 2 Laboratorio de Inflamación y Cáncer, Departamento de Química Biológica, Facultad de Ciencias Exactas y Naturales, Universidad de Buenos Aires, Buenos Aires, Argentina; 3 CONICET-Universidad de Buenos Aires. Instituto de Química Biológica de la Facultad de Ciencias Exactas y Naturales (IQUIBICEN), Buenos Aires, CP1428 Argentina; 4 Unidad de Transferencia Genética, Instituto de Oncología “Angel H Roffo”, Universidad de Buenos Aires, Buenos Aires, Argentina; 5 Department of Translational Molecular Pathology, The University of Texas MD Anderson Cancer Center, Houston, TX 77030 USA

**Keywords:** Cancer, Cancer metabolism

## Abstract

Prostate cancer (PCa) that progresses after androgen deprivation therapy (ADT) remains incurable. The underlying mechanisms that account for the ultimate emergence of resistance to ADT, progressing to castrate-resistant prostate cancer (CRPC), include those that reactivate androgen receptor (AR), or those that are entirely independent or cooperate with androgen signaling to underlie PCa progression. The intricacy of metabolic pathways associated with PCa progression spurred us to develop a metabolism-centric analysis to assess the metabolic shift occurring in PCa that progresses with low AR expression. We used PCa patient-derived xenografts (PDXs) to assess the metabolic changes after castration of tumor-bearing mice and subsequently confirmed main findings in human donor tumor that progressed after ADT. We found that relapsed tumors had a significant increase in fatty acids and ketone body (KB) content compared with baseline. We confirmed that critical ketolytic enzymes (ACAT1, OXCT1, BDH1) were dysregulated after castrate-resistant progression. Further, these enzymes are increased in the human donor tissue after progressing to ADT. In an *in silico* approach, increased *ACAT1, OXCT1, BDH1* expression was also observed for a subset of PCa patients that relapsed with low *AR* and *ERG* (ETS-related gene) expression. Further, expression of these factors was also associated with decreased time to biochemical relapse and decreased progression-free survival. Our studies reveal the key metabolites fueling castration resistant progression in the context of a partial or complete loss of AR dependence.

## Introduction

Androgen signaling inhibition remains the mainstay, and patients are treated similarly despite the known heterogeneity of responsiveness. The underlying mechanisms that account for the ultimate emergence of resistance to androgen deprivation therapy (ADT), progressing to castrate-resistant prostate cancer (CRPC), and to second generation androgen blockade, include those that reactivate androgen receptor (AR) signaling despite strong inhibition (e.g., AR amplification and/or mutation), or those that are entirely independent or cooperate with androgen signaling to underlie PCa progression [1].

It is well known that oncogenic alterations modify the metabolic program of cancer cells [2]. Mammalian cells use two main pathways to generate energy in the form of adenosine triphosphate (ATP) from glucose: oxidative phosphorylation in the mitochondria, with CO_2_ and H_2_O as final products, and glycolysis in the cytoplasm, yielding lactate. It is known that during neoplastic transformation, glucose is metabolized to lactate even in the presence of oxygen, the Warburg effect [3, 4].

The hypoxic conditions, commonly observed in tumors, force cancer cells to adapt and to exploit alternative fuel sources [5, 6]. In PCa, the metabolic pathways of greatest relevance in this context seem to be fatty acid (FA) and glutamine metabolism [7]. In a meta-analysis of clinical metabolic profiling of 136 cancer cohorts including blood, tissue, and urine samples, ketone bodies (KB) (known products of FA metabolism), and in particular β-hydroxybutyrate (BHBA), were identified among the most upregulated metabolites compared with normal specimens [8]. KB metabolism provides substrate availability for lipogenesis, critical for tumor biomass expansion. Further, gain of this lipogenic conduit is associated with shorter patient survival and greater tumor burden [9]. Expression of mitochondrial acetyl-CoA acetyltransferase (ACAT1) has been associated with aggressive PCa and biochemical recurrence after ADT [10], indicating that ketogenesis/ketolysis may also play a role in PCa progression.

The intricacy of energy metabolism pathways associated with neoplastic transformation and cancer progression is partially responsible for the extensive gaps in our understanding of this phenotype, with an obvious negative impact on our ability to harness them for clinical purposes. Progress in comprehending changes in the metabolic program during PCa progression has also been hampered by a lack of models, representative of the clinical spectrum and biologic complexity of PCa. Patient-derived xenografts (PDXs) have been developed and have led to therapeutically relevant approaches [11–14]. It is imperative to harness these available tools to dissect how this metabolic plasticity orchestrates progression and metastasis.

In this work, using PDXs that mimic the response of the human donor to ADT in a well-controlled study, we performed for the first time a comprehensive metabolomic analysis of PCa PDXs that relapsed following castration. We discovered a metabolic shift from high glycolytic activity to exacerbated KB metabolism, indicating that a subpopulation of CRPCs that progress with partial or complete loss of AR dependence are fueled by KB. We confirmed that expression of critical ketolytic enzymes was significantly augmented after castration-resistant progression in both the PDX tumor and its human donor tissue. Further, we assessed the expression of these enzymes in a subset of PCa patients that relapse and with metastatic disease and also analyzed the correlation with biochemical relapse and progression-free survival.

## Results

### PDXs mimic short- and long-term response to ADT in human donor

To model the response of PCa to castration we used a PCa PDX, MDA PCa 183, derived from a bone metastasis in a treatment naïve patient. A genomic characterization previously performed in the MDA PCa 183 tumors (whole genome, exome, and transcriptome sequencing) identified *PTEN* homozygous deletion, *TMPRSS2/ERG* and *TP53*/*SCFD1* rearrangements, and *PLK1* and *ERG* outlier expressions [15]. Furthermore, this PDX expresses wild-type AR and ERG, as assessed by IHC [15, 16].

**Fig. 1:**
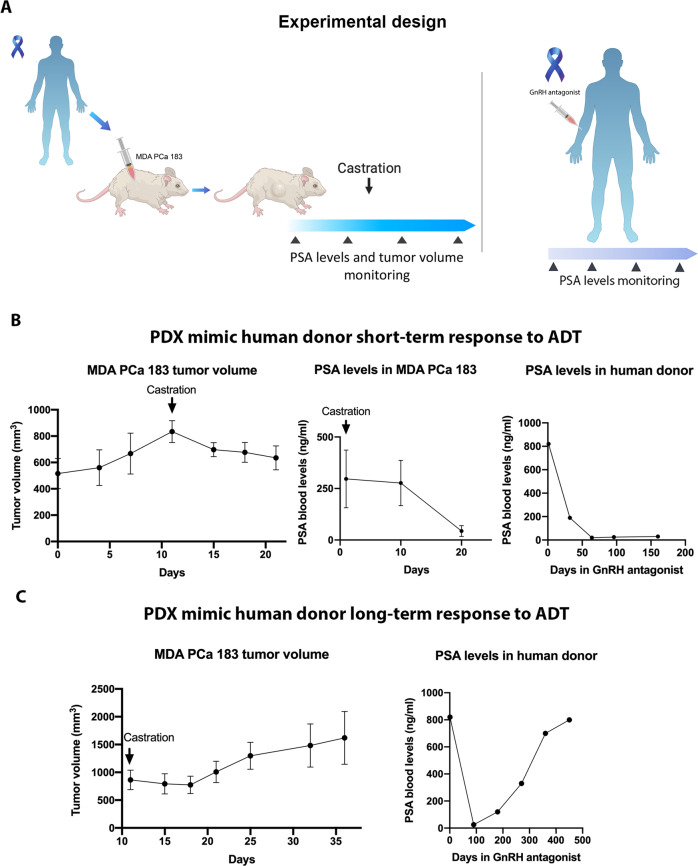
PDXs mimic response to ADT. **A** Schematic representation of subcutaneous injection of MDA PCa 183 (PCa bone metastasis) in CB17 SCID mice followed by castration when tumors reached a volume of 500 mm^3^ or higher (left panel) and human donor of MDA PCa 183 treated with ADT using GnRH antagonist (right panel). **B**, **C** Tumor volume and PSA levels of MDA PCa 183 subcutaneous tumors in mice and PSA levels assessed in response to androgen ablation in human donor of MDA PCa 183. Data are represented as mean ± SD.

We subjected mice bearing MDA PCa 183 tumors to surgical castration (Fig. 1A) and found a significant reduction in tumor volume with a concomitant drop in PSA blood levels (Fig. 1B). An analysis of tumor volume before and after castration demonstrated that there are statistically significant differences in the tumor growth slopes in intact vs castrated mice [17]. In parallel, the response of the patient donor of MDA PCa 183 to a gonadotropin-releasing hormone (GnRH) antagonist (Fig. 1A) was reflected by a drop in PSA blood levels (Fig. 1B) similar to the findings in MDA PCa 183 after castration, accompanied by improvement of symptoms associated with tumor burden.

In a long-term follow up we observed that a proportion of MDA PCa 183 tumors in castrated mice relapsed (Fig. 1C). Similarly, the patient response after four months undergoing ADT had evidence of disease progression according to an assessment of PSA levels (Fig. 1C), worsening of bone scan findings and bone metastasis-associated pain.

### Modeling and characterization of PCa response to castration

**Fig. 2:**
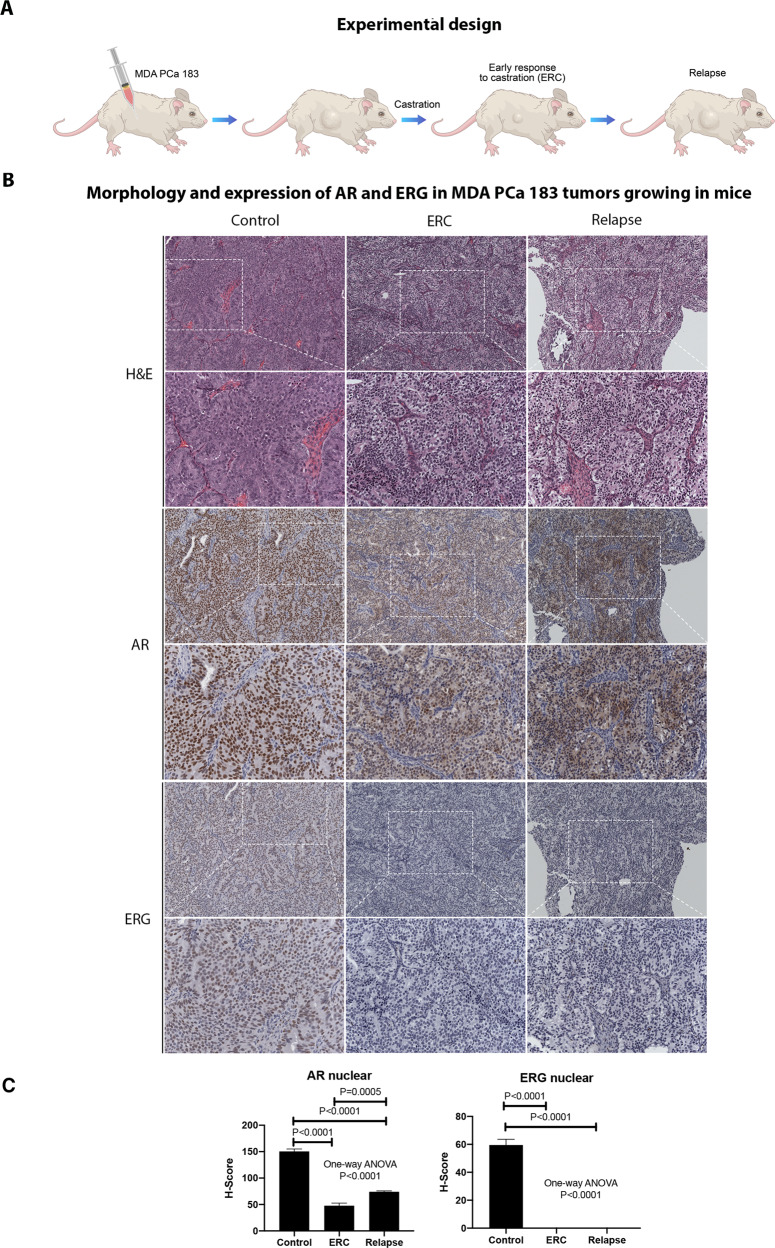
AR and ERG expression in MDA PCa 183 tumors in progression to castration. **A** Schematic representation of response to castration in mice bearing MDA PCa 183 tumor. **B** Representative photomicrograph images of MDA PCa 183 tumors (Control [*n* = 5], ERC [*n* = 6], and Relapse [*n* = 3]) sections immunostained with AR and ERG and (**C**) corresponding IHC quantification. Magnification 200X. Data are represented as mean ± SD. One-way ANOVA followed by Tukey’s multiple comparisons test was used to asses statistical significance (*P* < 0.05). ERC early response to castration, AR androgen receptor, ERG ETS-related gene.

To model PCa response to castration we obtained MDA PCa 183 tumors growing in intact mice (Control), those harvested 10 days after mice castration (when serum testosterone reached castration levels; early response to castration [ERC]) and at relapse (Relapse) (Fig. 2A). As depicted in Fig. 2B, MDA PCa 183 shows an adenocarcinoma tumor densely packed before castration (Control). At ERC and Relapse tumors seem less dense.

IHC analysis revealed that MDA PCa 183 tumors growing in control mice expressed high nuclear AR in nearly all cells, no cytoplasmic staining was observed. ERG staining was present in the nuclei of all cells (Fig. 2B, C). At ERC, where tumor growth was halted, AR nuclear expression was significantly lower (*P* < 0.0001), with heterogeneous nuclear localization and scarce cytoplasmic staining; while ERG reactivity was negative (*P* < 0.0001) (Fig. 2B, C). Likewise, in relapsed tumors AR expression remained low compared with control (*P* < 0.0001), with cytoplasmic and nuclear localization, and ERG staining remained negative (*P* < 0.0001) (Fig. 2B, C). These results suggest that MDA PCa 183 progressed to castration independently of AR signaling or by activating non-canonical AR signals.

We furthered our study performing an RNA-Seq analysis of 37 CRPC MDA PCa PDXs, [16, 18] focusing on AR and AR downstream targets’ gene expression (*ERG*, *TMPRSS2*, *KLK3*, *CAMKK2*, *NKX3.1*, *FKBP5*, *PGC*, *PMEPA1*). In accordance, results showcased that 42.8% of PDXs progressed with downregulated AR and the analyzed AR downstream targets (Fig. S1).

### Metabolomic shifts in PCa response to castration

**Fig. 3:**
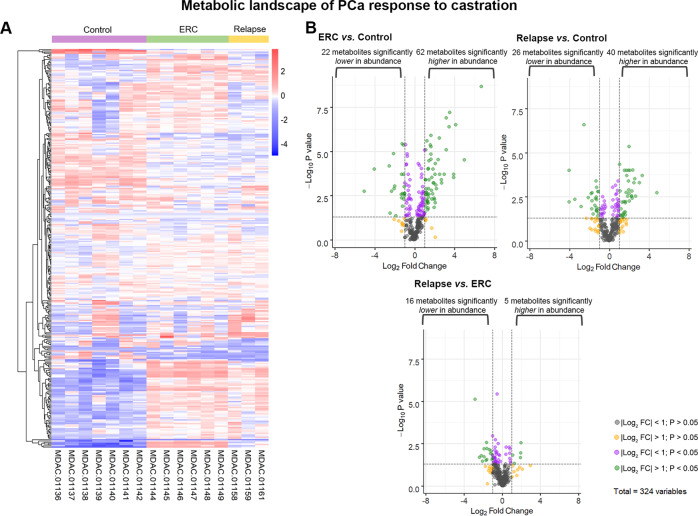
Metabolic shift of MDA PCa 183 tumors in progression to castration. **A** Heatmap depicting metabolites significantly higher or lower in Control (*n* = 7), ERC (*n* = 6) and Relapse (*n* = 3) tumors. Raw counts were rescaled to set the median equal to 1 and expressed as imputed normalized counts for each metabolite (ScaledImpData). Log of the ScaledImpData was plotted. Relative abundance was color labeled (red to blue). **B** Volcano plots depict the 324 metabolites profiled. Significant differential abundance (*P* value <0.05, |Log_2_ fold change| > 1) of higher and lower metabolites is represented for each comparison as green dots. Welch’s two-sample *t-*test was used to calculate statistical significance. NS not significant, Log_2_FC Log_2_ fold change. Grey dots: |Log_2_ fold change| < 1 and *P* value >0.05; yellow dots: |Log_2_ fold change| > 1 and *P* value >0.05; purple dots: |Log_2_ fold change| < 1 and *P* value <0.05; green dots: |Log_2_ fold change| > 1 and *P* < 0.05.

With the goal of understanding the metabolomic changes associated with PCa progression under ADT and identify the putative metabolites fueling CRPC, we used metabolomics techniques and bioinformatics tools. Briefly, tumor samples obtained from Control, ERC, and Relapse groups were subjected to UPLC-QTOF-MS/MS. The relative abundance of metabolites in Control, ERC, and Relapse tumors are depicted in the heatmap (Fig. 3A, Table S1). 324 named metabolites were profiled (Fig. 3B). The comparison among groups using Welch’s two-sample *t-*test identified the following changes: 84 metabolites exhibited significant differential abundance (|Log2 fold change| > 1, *P* value <0.05) when comparing ERC vs. Control tumors (22 lower and 62 higher); 66 in Relapse vs. Control tumors (26 lower and 40 higher); and 21 in Relapse vs. ERC tumors (16 lower and 5 higher) (Fig. 3B).

**Fig. 4:**
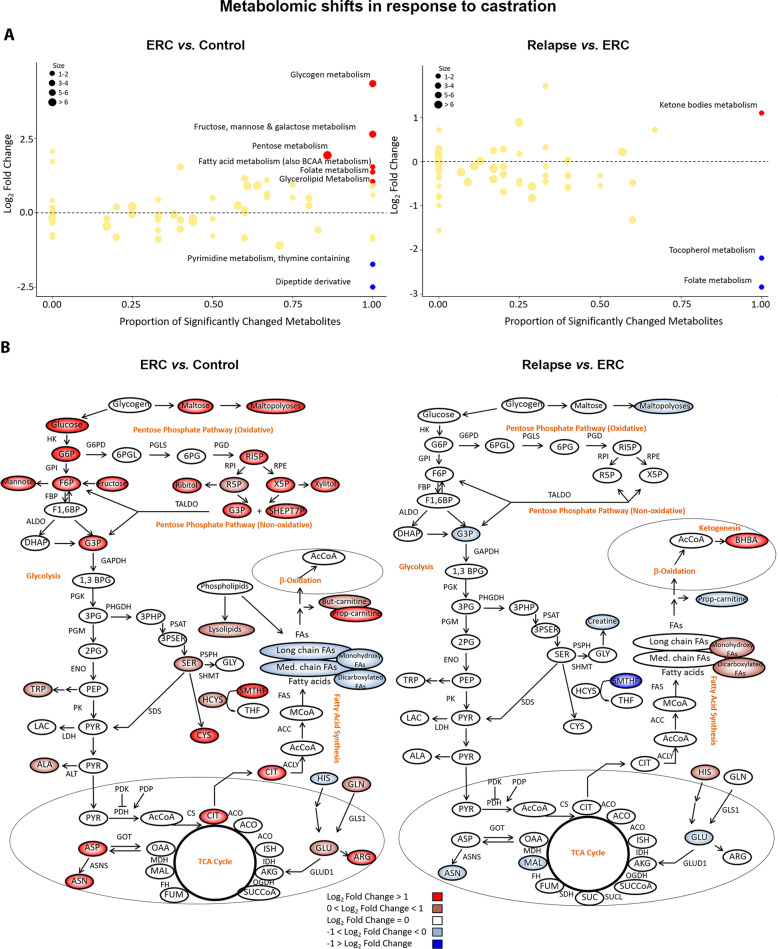
Pathway-based analysis of metabolic changes upon comparison of Relapse vs. ERC and ERC vs. Control groups. **A** Scatter plots depicting altered pathways according to the metabolites increased and decreased in each comparison using Welch’s two-sample *t*-test. The *x*-axis indicates the proportion of metabolites in a pathway that are significantly changed (both increased and decreased) in the comparison. The *y*-axis plots the average Log_2_ fold change of these metabolites. A proportion of significantly changed metabolites of 0.8 and a |Log2 fold change| > 1 were set as thresholds for pathways enrichment. **B** Metabolic changes of central carbon metabolism in ERC (*n* = 6) vs. Control (*n* = 7) or Relapse (*n* = 3) vs. Control (*n* = 7) comparisons. Metabolites are labeled as color-coded ovals (red: upregulated; blue: downregulated). Color corresponds to the Log_2_ fold changes within a comparison. Enzymes for individual chemical reactions are denoted next to the arrows connecting two metabolites. G6P glucose 6-phosphate, F6P fructose 6-phosphate, F1,6BP fructose 1, 6-bisphosphate, DHAP dihydroxyacetone phosphate, G3P glyceraldehyde 3-phosphate, 1,3BPG 1,3-bisphosphoglycerate, 3PG 3-phosphoglycerate, 2PG 2-phosphoglycerate, PEP phosphoenolpyruvate, TRP tryptophan, PYR pyruvate, LAC lactate, ALA alanine, 3PHP 3-phosphohydroxypyruvate, 3PSER 3-phosphoserine, SER serine, AcCoA acetyl-CoA, ISC, isocitrate, CIT citrate, ACO cis-aconitate, AKG alpha-ketoglutarate, SUCCoA succinyl-CoA, SUC succinate, FUM fumarate, MAL malate, OAA oxaloacetate, GLN glutamine, GLU glutamate, CYS cysteine, GLY glycine, MCoA malonyl-CoA, HCYS homocysteine, 6PGL 6-phosphogluconolactone, 6PG 6-phosphogluconate, R5P ribose 5-phosphate, RI5P ribulose 5-phospate, X5P xylulose 5-phosphate, ASP aspartate, ASN asparagine, ARG arginine, HIS histidine, 5MTHF levomefolic acid, THF tetrahydrofolate, BHBA β-hydoxybutirate, SHEPT7P sedoheptulose-7-phosphate, ERC early response to castration.

Subsequently, we investigated the pathways associated with the abovementioned metabolic profiles. For these analyses we focused on comparing Relapse vs. ERC and ERC vs. Control. In the first comparison, the tumor-bearing mice of both groups were castrated, but ERC tumors were not growing. Therefore, metabolites that increase in relapsed tumors (as compared with ERC) reflect changes associated with progression under ADT. In the second comparison, ERC vs. Control, differences in metabolite abundance reflect adjustment of ERC tumors to castration. We plotted the proportion of metabolites in the sub-pathways that changed significantly for each comparison (*x*-axis), vs. the average Log_2_ (Fold Change) of these metabolites (*y*-axis) (Fig. 4A, Fig. S2A). For the ERC vs. Control comparison, the most significant elevated pathways were: (1) glycogen, (2) fructose, mannose & galactose, (3) pentose, (4) fatty acid (also branched chain amino acid [BCAA]), (5) folate, and (6) glycerolipid metabolism, whereas pyrimidine (thymine containing) and dipeptide derivative were downregulated pathways (Fig. 4A, left panel). Interestingly, the most significant pathway elevated in Relapse vs. ERC was involved in KB metabolism, whereas the decreased pathways were involved in tocopherol and folate metabolism (Fig. 4A, right panel). In particular, 3-hydroxybutyrate in the KB metabolism category had a >2-fold induction in Relapse vs. ERC tumors. On the other hand, alpha-tocopherol and 5MeTHF (5-methyltetrahydrofolate) were significantly downregulated in this comparison (Fig. S2B).

To better understand the metabolic alterations that occur upon relapse, we constructed a metabolic map detailing the shift in abundance of central carbon metabolites from Control to ERC and from ERC to Relapse (Fig. 4B). In the case of Relapse vs. ERC, we observed that the only increased metabolites were β-hydroxybutyrate (BHBA), histidine, and the following fatty acids derivatives: dicarboxylate FA (tetradecanedioate and hexadecanedioate) and monohydroxy FA (13-HODE + 9-HODE) (Fig. 4B, right panel). In this comparison, it is noteworthy to mention the reduction in abundance of metabolites in the upper glycolysis and pentose pathways (PPP), as opposed to ERC vs. Control, where we observed a significant increase in metabolites involved in glycogen, pentose, mannose, fructose, and galactose metabolism (Fig. 4B, left panel). ERC activation of metabolites in upper glycolysis, including glucose 6-phosphate (G6P) and fructose 6-phosphate (F6P) (>2-fold increases in abundance), suggests increased glucose uptake. In addition, the shift toward the PPP observed in ERC vs. Control suggests increased shunting into the PPP to produce ribose 5-phosphate (R5P) and NADPH. Lysolipids are also increased to a lesser extent with concomitant reduction in FA, thus probably diverting energy production for biosynthesis of membranes and storage, favoring tumor dormancy (Fig. 4B, left panel).

### Dysregulated ketogenic/ketolytic enzymes in tumor-bearing PDX and human donor during PCa progression

**Fig. 5:**
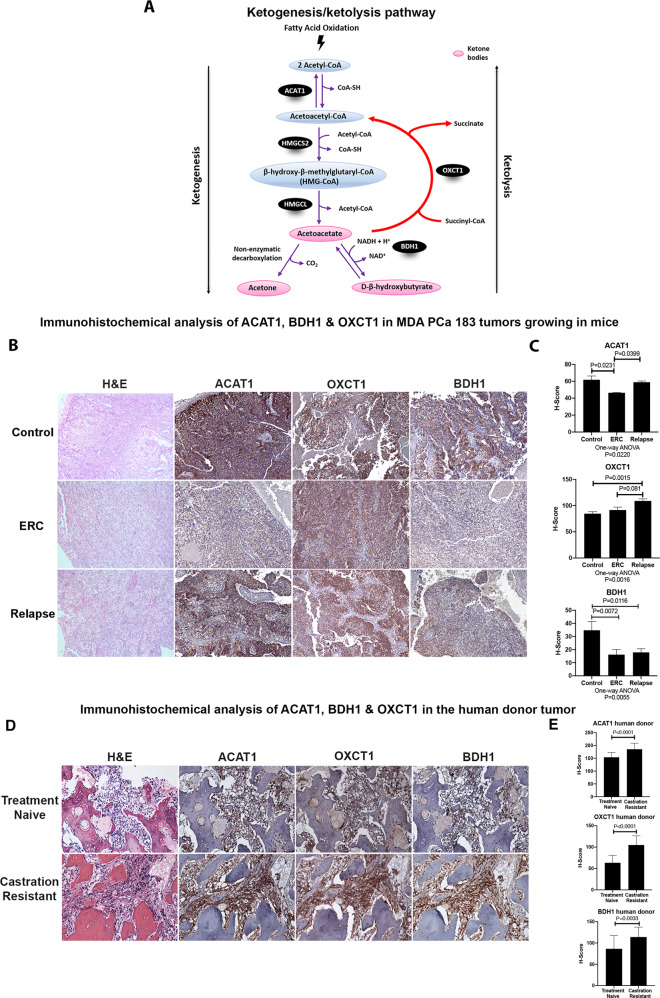
Upregulation of ketogenic/ketolytic enzymes in preclinical and clinical progression of PCa. **A** Schematic representation of ketogenesis/ketolysis pathway. The three ketone bodies are marked within a pink oval. Enzymes are depicted in black ovals and the other named metabolites in blue ovals. **B** Representative photomicrograph images of sections of MDA PCa 183 tumors growing in mice and (**C**) corresponding IHC quantification (Control [*n* = 5], ERC [*n* = 6] and Relapse [*n* = 3]). Magnification 100X. Data are represented as mean ± SD. One-way ANOVA followed by Tukey’s multiple comparisons test was used to assess statistical significance (*P* < 0.05). **D** Representative photomicrograph images of sections of samples of the corresponding human donor of MDA PCa 183 before ADT (treatment naive) and after progression (castration resistant) and (**E**) corresponding IHC quantification. Magnification 200X. Data are represented as mean ± SD. Two-tailed *t*-test was used to assess statistical significance (*P* < 0.05). Samples were stained with H&E and immunostained for ACAT1, OXCT1, and BDH1. ERC early response to castration, H&E Hematoxylin and eosin, ACAT1 acetyl-CoA acetyltransferase, OXCT1 3-oxoacid CoA-transferase 1, BDH1 3-Hydroxybutyrate Dehydrogenase 1, HMGCS2 hydroxy-methylglutaryl-CoA synthase 2, HMGCL hydroxy-methylglutaryl-CoA lyase.

Our previously described findings showcase KB as one of the main metabolites highly increased during PCa growth under ADT. Therefore, we sought to evaluate enzymes involved in ketogenesis/ketolysis, namely ACAT1, 3-Hydroxybutyrate Dehydrogenase 1 (BDH1) and 3-oxoacid CoA-transferase 1 (OXCT1). ACAT1, BDH1 are reversible enzymes of the ketogenic/ketolytic pathway (Fig. 5A), allowing either the formation of KB or conversion to acetyl-CoA. However, OXCT1 catalyzes the irreversible reaction that transforms succinyl-CoA to acetoacetyl-CoA in most extrahepatic tissues [19]. In line with the metabolomics data, we confirmed by IHC analysis the increased expression of the ketogenic enzyme ACAT1 in Relapse vs. ERC (Fig. 5B, C). BDH1 showed decreased expression when comparing ERC vs. Control, but no significant difference between ERC and Relapse. In the case of OXCT1, there was no difference between Control and ERC, but a marginal significant increase (*P* = 0.081) was observed when comparing Relapse *vs*. ERC (Fig. 5B, C). OXCT1 is an extrahepatic enzyme, which only participates in ketolysis (Fig. 5A), hence, the increased levels of OXCT1 may suggest utilization of KB as fuel by relapsed tumors. Most importantly, when we studied the expression of ACAT1, OXCT1, and BDH1 in the human donor tumor of MDA PCa 183, we detected significant increased expression of these enzymes in PCa progressing to ADT as compared with the untreated tumor of the same patient (Fig. 5D, E, *P* < 0.0001, *P* < 0.0001, *P* = 0.0033, respectively). Accordingly, MDA PCa 203, the longitudinal sample of MDA PCa 183, derived from the same human donor after relapse (castration resistant) exhibited significant increased expression of these enzymes compared with MDA PCa 183 (Fig. S3A). These results support the concept that the changes observed in our PDX were predictive of the changes that we identified in the human donor.

Further, we assessed the expression of the ketogenic enzymes hydroxy-methylglutaryl-CoA synthase 2 (HMGCS2), hydroxy-methylglutaryl-CoA lyase (HMGCL) and the KB transporter monocarboxylate transporter 2 (SLC16A7) (Fig. S3B). IHC analysis showcased increased staining for HMGCS2 both, in ERC vs. Control (*P* = 0.0049) and in Relapse vs. ERC (*P* = 0.02222). On the other hand, HMGCL was significantly decreased (*P* = 0.0377) in ERC vs. Control; however, no statistical differences were observed for ERC vs. Relapse. Taking into consideration that HMGCL is an irreversible enzyme of the pathway, this might indicate that ketogenesis is not favored in relapsed tumors. Regarding the SLC16A7 transporter, a slight significant decrease was observed in Relapse vs. ERC tumors (*P* = 0.0286) (Fig. S3B).

Together, these data reflect a metabolic switch that accompanies relapse, where a tilt in the balance towards ketolysis is observed. This tumor cell energetic plasticity may denote an unseen landmark for early relapse in PCa.

### Analysis of ketogenic/ketolytic enzymes as risk predictors of clinical outcome in CRPC

**Fig. 6:**
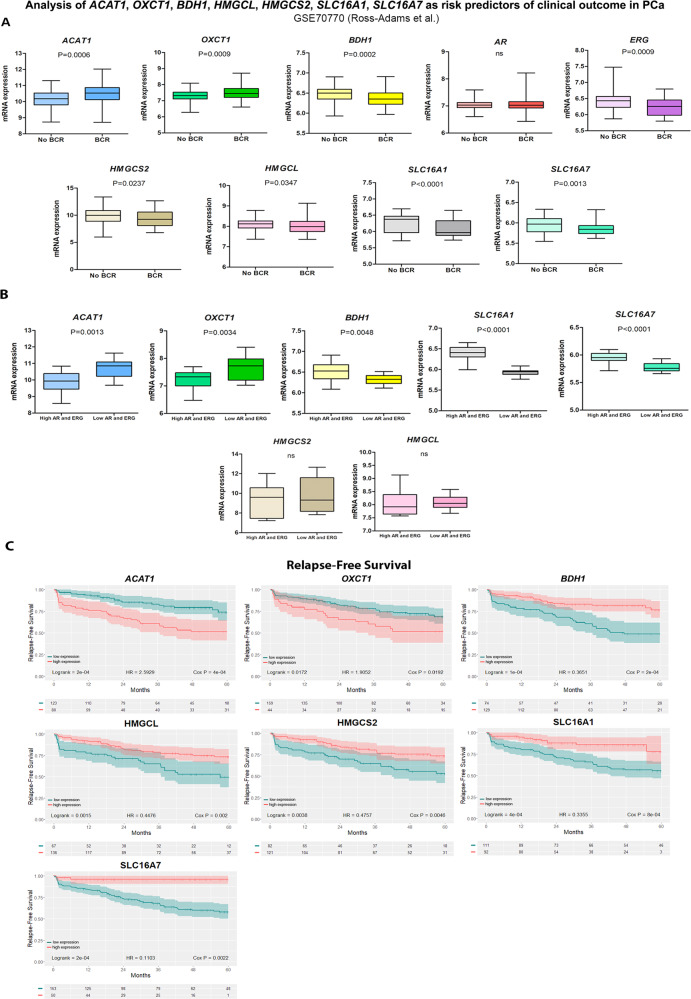
Bioinformatics analysis of ketogenic/ketolytic enzymes and MCT transporters as risk predictors of clinical outcome in CRPC. **A** Gene expression analysis for *ACAT1*, *OXCT1*, *BDH1*, *AR, ERG, HMGCS2, HMGCL, SLC16A1* and *SLC16A7* comparing PCa patients that underwent radical prostatectomy and biochemically relapsed (BCR; *n* = 59) vs. those that did not relapse (No BCR; *n* = 145) in the Ross-Adams dataset (GSE70770; *n* = 204). Data are presented as box-and-whisker plots (min-max error bars). *t*-test was used to assess statistical significance. **B** Gene expression analysis in PCa patients with BCR categorized by *ERG/AR* status (high expression *n* = 16; low expression *n* = 15). Data are presented as box-and-whisker plots (min-max error bars). *t*-test was used to assess statistical significance. **C** Kaplan–Meier (KM) curves for relapse-free survival (RFS) in months for PCa patients with low (green) or high (red) *ACAT1*, *OXCT1*, *BDH1, HMGCS2, HMGCL, SLC16A1* and *SLC16A7* expression in the Ross–Adams dataset. Log-rank test and Cox proportional hazard model regression were employed to assess statistical significance. All comparisons considered low expression patients as the reference group. HR: hazard ratio. Statistical significance: *P* < 0.05.

As mentioned above, ACAT1, OXCT1 and BDH1 are critical in ketone metabolism. To further ascertain the implications of these factors in CRPC, we mined the Ross-Adams dataset (GSE70770, *n* = 204). In-depth analysis of PCa patients that had undergone radical prostatectomy, revealed significant higher gene expression profiles for *ACAT1* (*P* = 0.0006) and *OXCT1* (*P* = 0.0009), in PCa patients that biochemically relapsed (BCR) within a five-year follow-up period, as compared with patients that did not relapse in the same time-period (No BCR) (Fig. 6A). However, *BDH1* expression levels were higher in non-recurrent tumors compared with BCR (*P* = 0.0002). When extending the analyses to the ketogenic enzymes *HMGCL* & *HMGCS2* and the KB transporters *SLC16A1* and *SLC16A7*, increased expression levels were observed for all of them in non-recurrent tumors compared with BCR (*P* = 0.0347, *P* = 0.0237, *P* < 0.0001 and *P* = 0.0013, respectively). Given that in our preclinical studies, relapsed tumors did not express ERG and AR expression was low (Fig. 2B), we also assessed *ERG* and *AR* expression in this dataset. Results indicated that BCR patients had reduced *ERG* levels (*P* = 0.0009) and no significant changes were found for *AR* (Fig. 6A). In an independent analysis, when taking into consideration only patients that relapsed, those with low *AR* and *ERG*, presented high *ACAT1* and *OXCT1* expression (*P* = 0.0013 and *P* = 0.0034, respectively) and low *BDH1* (*P* = 0.0048), *SLC16A1* (*P* < 0.0001) and *SLC16A7* (*P* < 0.0001) (Fig. 6B). These results ascertain our IHC observations on the augmented expression of ACAT1 and OXCT1 and diminished expression of SLC16A7 in the preclinical model. Further, the results on ACAT1 and OXCT1 are in accordance with the IHC performed on the human donor upon relapse and the longitudinal sample of MDA PCa 183, derived from the same human donor after relapse. However, discrepancies arise for BDH1. Of note, this protein presented no significant changes in Relapse vs. ERC in our preclinical model, appeared significantly increased in the human donor and longitudinal sample of MDA PCa 183 upon relapse, while it seemed downregulated at the transcriptional level in the Ross-Adams dataset. We cannot discard differential regulation at the translational or post-translational level that justify the accumulation of the protein which was detected by IHC (Fig. 5B–E) [20].

**Fig. 7:**
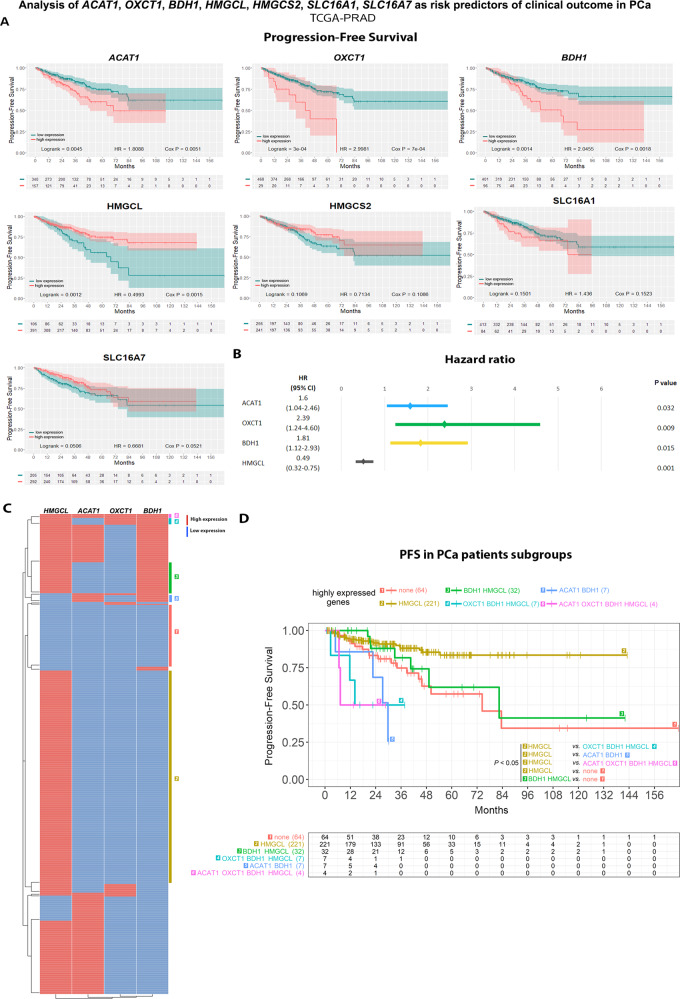
Bioinformatics analysis of ketogenic/ketolytic enzymes and MCT transporters as risk predictors of clinical outcome in CRPC. **A** Kaplan–Meier (KM) curves for progression-free survival (PFS) in months for PCa patients with low (green) or high (red) *ACAT1, OXCT1, BDH1, HMGCL, HMGCS2, SLC16A1* and *SLC16A7* expression in the TCGA-PRAD dataset (*n* = 497). Log-rank test and Cox proportional hazard model regression were employed to assess statistical significance. **B** Multivariable Cox proportional hazard model regression analysis for *ACAT1*, *OXCT1*, *BDH1* and *HMGCL* presented as forest plots for PFS. **C** Heatmap depicting low (blue) or high (red) *ACAT1*, *OXCT1*, *BDH1* and *HMGCL* mRNA expression for PCa patients according to the TCGA-PRAD dataset. **D** KM curves for PFS in months for PCa patients subgroups with different expression levels of *ACAT1*, *OXCT1*, *BDH1* and *HMGCL* in TCGA-PRAD: [1] low *ACAT1*, *OXCT1, BDH1* and *HMGCL* expression (*n* = 64); [2] low *ACAT1*, low *OXCT1, low BDH1* and high *HGMCL* expression (*n* = 221); [3] high *BDH1* and *HMGCL* and low *ACAT1* and *OXCT1* expression (*n* = 32), [4] low *ACAT1* and high *OXCT1*, *BDH1* and *HMGCL* expression (*n* = 7), [5] high *ACAT1* and *BDH1* and low *OXCT1* and *HMGCL* expression (*n* = 7); [6] high *ACAT1, OXCT1*, *BDH1* and *HMGCL* expression (*n* = 4). The table indicates the number of patients assessed every 12 months. Log-rank test was employed to assess statistical significance. HR: hazard ratio. Statistical significance: *P* < 0.05.

Next, we evaluated the relapse-free survival time (RFS) in PCa patients expressing high or low *ACAT1, OXCT1, BDH1, HMGCL, HMGCS2, SLC16A1* and *SLC16A7*. Results demonstrated significant association of high *ACAT1* and *OXCT1* expression with shorter RFS (Fig. 6C). The opposite was observed for *BDH1*, *HMGCL*, *HMGCS2, SLC16A1* and *SLC16A7* (Fig. 6C). Interestingly, when performing the same analysis in the TCGA-PRAD dataset (*n* = 497), high expression of *ACAT1, OXCT1* and *BDH1* was associated with shorter progression-free survival (PFS) (Fig. 7A), while higher levels of *HMGCL* were associated with a longer PFS time. Indeed, these results support the preclinical/clinical data about ACAT1 and OXCT1 expression as risk predictors upon relapse and evidence the discrepancies regarding BDH1. In addition, increased HMGCL which could favor ketogenesis, presents similar pattern regarding the increase in PFS and RFS.

To validate the potential of these molecules to improve risk stratification in PCa patients and in order to assess variable independence, multivariable analysis was performed including only those genes that displayed a significant HR in the univariable analysis in the TCGA-PRAD dataset. Results showed that *ACAT1, OXCT1, BDH1* and *HMGCL* could be independent predictors of disease progression (*ACAT1* [*P* = 0.032], *OXCT1* [*P* = 0.009], *BDH1* [*P* = 0.015] and *HMGCL* [*P* = 0.001]) (Fig. 7B). Next, we grouped PCa patients based on high or low *ACAT1, OXCT1, BDH1* and *HMGCL* gene expression level. We categorized the patients in groups according to the combinatorial expression of these four genes (Fig. 7C). We then performed a PFS analysis of these patient subgroups. Figure 7D depicts the Kaplan-Meier curve for the subgroups showcasing a significant difference in PFS. All comparisons are shown Fig. S4. Patients with high expression of *HMGCL* (group 2) and high expression of *BDH1* and *HMGCL* (group 3) had significantly increased PFS time compared with patients in group 1 (low expression for all assessed genes *ACAT1*, *BDH1*, *OXCT1* & *HMGCL*) (*P* < 0.05 for all comparisons) (Fig. 7C). In addition, group 2 had an increased PFS compared with groups 4 (high *OXCT1*, *BDH1* & *HMGCL*), 5 (high *ACAT1* & *BDH1*) and 6 (high expression for all assessed genes). These results evidence the relevance of these genes in the risk of progression of PCa patients, especially the increased risk observed with high expression for *ACAT1* and *OXCT1*. Of note, higher levels of *HMGCL* appear to improve the PFS in patients with no elevated levels of ketolytic enzymes.

In parallel, the same analysis was performed on the SU2C dataset, which contains metastatic PCa samples. We found that only high expression of *ACAT1, HMGCL, SLC16A1*, and *SLC16A7* correlates with a shorter overall survival (*P* = 0.0403, 0.0387, 0.0002 and 0.0395, respectively) (Fig. S5A). The multivariable analysis showed that only *SLC16A1* behaved independently (*P* = 0.005) (Fig. S5B). When categorizing patients in groups according to the combinatorial expression of these four genes associated with a shorter survival time in the univariable analysis (Fig. S5C), we observed that those patients with a combined high expression of *HMGCL* and *SLC16A1* (group 2) had a worse outcome compared with those patients with high expression only for *HMGCL* (group 1) or with those patients with low expression levels for all genes (group 3) (Fig. S5D). Non-statistically significant comparisons are not depicted in the figure. These results favor a worst outcome with high levels of the KB transporter despite the presence of *HMGCL*.

Furthermore, we assessed the expression of the ketogenic/ketolytic enzymes and the monocarboxylate transporters at the transcriptomic level in human samples of primary and metastatic PCa using the dataset GSE74685, which contains 149 samples of metastasis and 14 primary PCa tumors. The analysis showed that the expression of *ACAT1*, *BDH1* and *SLC16A1* was significantly higher in bone metastatic samples when compared with the primary tumor site (*P* = 0.0042, *P* = 0.0091, and *P* = 0.0006, respectively) (Fig. S6A). In addition, lymph node metastasis had an increased level of *ACAT1* expression (P = 0.0429), while *HMGCL* was higher in liver metastasis compared with primary tumors (*P* = 0.0274) (Fig. S6A). No significant differences were detected for *OXCT1*, *HMGCS2* and *SLC16A7* when comparing primary tumor samples against the different PCa metastatic sites.

In parallel, we used the data from GSE32269 [21] to compare the expression levels of the key ketogenic/ketolytic enzymes in 22 primary PCa (hormone-dependent) vs. 29 bone metastatic CRPC samples. We observed that the expression of *ACAT1* and the monocarboxylate transporter *SLC16A1* was higher in bone metastatic samples (*P* = 0.0281 and *P* = 0.0204, respectively) while the expression of *HMGCL* was lower in CRPC samples (*P* < 0.0001). No differences were detected for *OXCT1*, *BDH1, SLC16A7* and *HMGCS2* transcriptional levels (Fig. S6B).

Overall, results from the metastatic datasets continue to add evidence towards the critical role of ketolytic enzymes in PCa progression and the KB transporter SLC16A1 appears to be significantly increased in metastatic sites, pointing out to KB as a valid fuel source for progression.

## Discussion

In this work we are reporting a metabolic shift towards ketolysis that occurs in PCas that relapse after ADT. Furthermore, we have discovered that these metabolic alterations occur in cells that do not use the canonical AR signaling while progressing to ADT. Briefly we observed that relapsed tumors have an increase in KB content while tumor cells have reduced nuclear AR, increase of cytoplasmic AR and loss of ERG expression as compared with controls.

Reports evidence that metabolic changes are acquired during PCa progression and may underlie CRPC growth [22, 23]. To further understand metabolic changes that occur during progression we performed metabolomics analyses in well-established PDXs that mimic donor response to ADT in a well-controlled study, which is difficult to achieve with clinical samples. The MDA PCa models have already provided unique insights into the biology of PCa [16], including the discovery of: distinct classes of chromosomal rearrangements in PCa cells [24]; new therapeutic approaches for combination therapy targeting DNA damage response genes [25, 26]; new biological roles of genes in PCa [27]; new mechanisms underlying neuroendocrine differentiation [28]; molecular alterations commonly found in aggressive variant PCa [29]; fibroblast growth factor (FGF) axis in the pathogenesis of PCa bone metastasis [11, 14, 30, 31].

As mentioned earlier, the MDA PCa 183 PDX used to perform our studies expresses wild-type AR, and has *TMPRSS2/ERG* rearrangement and ERG outlier expressions [15]. However, when these tumors relapse, the change in AR subcellular localization and absence of ERG, indicate that these tumors progressed to castration without canonical AR signaling activation, reflecting progression with partial or complete loss of AR dependence.

Metabolomic analyses of MDA PCa 183 tumors after castration of tumor-bearing mice, showed a significant reduction in abundance of upper glycolysis and PPP metabolites, in Relapse vs. ERC as opposed to ERC vs. Control. ERC activation of metabolites in upper glycolysis, suggested increased glucose uptake. In addition, the shift toward the PPP observed in ERC vs. Control suggests increased shunting into the PPP to produce R5P and NADPH. These results depict that upon castration tumor cells activate upper glycolysis to survive and adapt.

Relapsed tumors had a significant increase in FA derivatives and BHBA (KB) compared with tumors at ERC. KB are high-energy mitochondrial fuels that can be converted back into acetyl-CoA and reutilized as an energy source [32, 33]. Upon relapse, at least in our experimental model and human donor, PCa cells did not show increased lactate levels that could evidence the Warburg effect. Thus, it does not seem as if lactate would be fueling their metabolism.

BHBA has been implicated in cancer by playing both metabolic and epigenetic roles, and exerting contrasting effects in tumor progression. This phenomenon, known as the “butyrate paradox”, has been discussed for butyrate and suggested to analogously occur for BHBA [34]. By inhibition of histone deacetylases and consequently, selective induction of negative regulators of cell cycle, BHBA/butyrate hinders proliferation of cancer cells favoring glucose usage (Warburg effect). However, low levels of BHBA/butyrate, due to its consumption as an oxidative fuel, instead of glucose/Warburg effect, has been shown to fail in modulating histone acetylation, and therefore, in tumor growth inhibition, resulting in a pro-tumorigenic effect [35]. In line with the latter findings, in this study, we observed a re-establishment of the levels of BHBA upon Relapse following ERC, compared with Control conditions (Fig S2 B), suggesting BHBA could drive CRPC progression via its dual function. Further, butyrate has been associated with induction of catalase, involved in protection from oxidative perturbations; transcription factors FOXO3a and PGC-1α and mitochondrial genes, that play a critical role in oxidative stress response; and genes with anti-inflammatory properties. In addition, butyrate downregulates expression of pro-inflammatory factors and is a well-known NF-κB inhibitor, resulting in decreased ROS levels and inflammation. On the contrary, BHBA was unsuccessful in reducing expression of pro-inflammatory genes and, even more, activated molecules of the NF-κB pathway and thus, oxidative stress and inflammation [36]. Therefore, it remains unclear up to what extent the properties observed for butyrate extend to BHBA and need further study.

Since KB can be produced by β-oxidation of FA [37], which were also found to increase in relapsed tumors, we cannot rule out that FA could be the main source of energy implicated in CRPC progression. Under this scenario, KB could be a product of β-oxidation and a biomarker of CRPC progression. However, we confirmed that the expression of critical ketolytic enzymes was significantly augmented after castration-resistant progression in MDA PCa 183 tumor. When assessing the expression of the ketogenic enzymes HMGCS2, HMGCL, the IHC analysis revealed increased HMGCS2 for ERC vs. Relapse but no statistical difference for HMGCL in the same comparison. Taking into consideration that HMGCL is an irreversible enzyme of the pathway, this might indicate that ketogenesis is not favored in relapsed tumors. Our discovery that the ketolytic enzyme enhancements were also found in the human donor tissue after progressing to ADT add relevance to our findings. Together, these data may indicate a tilt in the balance towards ketolysis upon progression.

Expression of ACAT1, one of the key enzymes involved in the conversion of KB into acetyl-CoA, has been associated with aggressive PCa [22] and PCa biochemical recurrence following ADT [10]. Thus, regardless of KB source of origin and because of the reported increase in ACAT1 in PCa and its association with relapse to ADT, KB appear as the high-energy fuel driving CRPC.

Most KB production occurs in the liver [37], however smaller amounts are generated in other tissues through altered expression of ketogenic enzymes [38, 39] or reversal of the ketolytic axis. Once the BHBA is incorporated by a tissue, it is converted back into acetoacetate by the same enzyme that generated it (BDH1). From there, the pathway of KB utilization diverges from the ketogenic synthetic pathway. CoA is donated by succinyl-CoA to acetoacetate, to form acetoacetyl-CoA, a reaction catalyzed by OXCT1, bypassing the essentially irreversible reaction catalyzed by hydroxy-methylglutaryl-CoA synthase 2 (HMGCS2). As OXCT1 is an extrahepatic enzyme [37], these different enzymatic routes of synthesis and utilization of KB, prevent a BHBA useless cycle in the liver, allowing this metabolite to travel to other tissues as energy source. Thus, the acetoacetyl-CoA can then be converted back to two acetyl-CoA and incorporated into the Krebs cycle for oxidation and ATP formation [37]. Of note, OXCT1 was significantly increased in the human donor progressing on ADT therapy (castrate resistant) and on MDA PCa 203, the longitudinal sample of MDA PCa 183, derived from the same human donor after relapse (castration resistant). This result may evidence increased ketolysis and subsequent acetyl-CoA formation, vital for amino-acids, ATP and FA synthesis, favoring tumor progression. However, this should be further assessed.

Interestingly, in our univariable time‐to‐recurrence analysis in the TCGA-PRAD dataset, a positive association of increased expression of *ACAT1*, *OXCT1* & *BDH1* and decreased *HMGCL & HMGCS2* with PCa progression was observed. When performing multivariable analyses, *ACAT1*, *OXCT1*, *BDH1* & *HMGCL* transcripts appear to behave independently when predicting risk of progression. Further, when regrouping PCa patients according to these transcript levels, results evidenced the increased risk of progression for PCa patients with high expression for *ACAT1* and *OXCT1*. Interestingly, higher levels of *HMGCL* appear to improve the PFS in patients with no elevated levels of ketolytic enzymes.

Reports have shown ACAT1 expression to predict recurrence in ERG-negative cases, whereas ERG-positive cases did not display any difference [10]. However, no reference was provided as to whether the ERG-negative cases presented a *TMPRSS2*/*ERG* translocation that had bypassed the AR signaling or simply did not present the translocation at all. For this reason, ACAT1 appears as an exploitable target to assess its implications accompanying PCa relapse to ADT associated with low or negative AR expression.

ACAT1 was proposed as a druggable target for cancer therapy since in its active phosphorylated tetrameric form, catalyzes the conversion of two acetyl-CoA to acetoacetyl-CoA and CoA and also acts as an upstream acetyl transferase for the pyruvate dehydrogenase complex, a decisive point between two paths: glycolysis and oxidative phosphorylation [40]. In many cancer types, active ACAT1 inhibits pyruvate dehydrogenase activity and deflects pyruvate to lactate production. However, our results demonstrated high expression of ACAT1 in Relapse vs. ERC tumors, with augmented KB but no increase in lactate production. Since KB appeared as the main route fueling energy during relapse to ADT, inhibition of ACAT1 may block this energy source rather than trigger lactate production in this type of tumor. Moreover, ACAT1 is common to both, the mevalonate pathway (biosynthesis of cholesterol, sterols, etc.) and KB production, hence targeting this enzyme appears to be promising for therapeutic intervention of CRPC.

Several studies have discussed the fact that a dietary restriction enables the reduction in tumor volume and growth rate [41], improves patients’ quality of life [42] and survival [43], and also increases radiotherapy and chemotherapy sensitivity [44, 45]. However, in light of the vast amount of metabolic data associated with different cancer cell metabolic phenotypes, it is worth mentioning that the efficacy of a ketogenic diet (KD) in potentiating anti-tumor effects still remains controversial. The key in determining the success of KD might rely in a combination between the availability of KB and the expression levels of ketogenic/ketolytic enzymes. A study by Zhang et al. [46] showed that xenograft tumors that derive from cancer cells with low expression levels of BDH1 and OXCT1 are more responsive to KD therapy, probably because they possess a lower ability to metabolize KB. High expression of these enzymes reflects opposite KD effects on cancer in different studies. It is clear that if a tumor is unable to process KB, the use of such diet may be an effective strategy for nutrient starvation and tumor suppression. However, several reports have shown variable KD efficacies, tearing up the strategy of using KD to cure cancer. KD has proven ineffective in lung cancer cell xenografts and also in hepatocellular carcinoma cells that employ KB for energy supply and cancer progress [47].

Our preclinical CRPC model, and the *in silico* PCa data showcase metabolic adaptations -presence of KB and reactivation of key ketone catabolic enzymes- associated with PCa progression. Hence, this subpopulation of PCas with high ketolytic enzymes may influence the effectiveness of a KD. Thus, assessing in combination both, the KB content and the expression levels of these enzymes, may be crucial in stratifying PCa patients for which a KD may prove successful.

Overall, our work has identified critical metabolic changes in early relapse following ADT of a subpopulation of PCa that progress with low AR/ERG. These metabolic changes may serve as the foundation to identify early biomarkers of CRPC progression in a larger study.

## Materials and methods

### MDA PCa 183 and 203 PDX generation

At MD Anderson, we established a program to develop PCa PDXs (the MDA PCa PDX series) using clinically annotated PCa specimens with the goal of more comprehensively modeling the complexity of PCa. The PDX MDA PCa 183 was developed in our laboratory from a bone marrow aspirate of a human male with treatment naïve metastatic adenocarcinoma [15, 16, 18] as described previously [16], and propagated as subcutaneous xenografts in six- to eight-week-old male CB17 SCID mice (Charles River Laboratories; Wilmington, MA, USA). The longitudinal sample of MDA PCa 183, MDA PCa 203, was developed from the same donor after its PCa relapsed [18], and was propagated as described above, using surgically castrated mice as the hosts.

### MDA PCa 183 growth in intact and castrated mice

Studies were conducted as described elsewhere [16]. Briefly, six- to eight-week-old male CB17 SCID mice (*n* = 20) were subcutaneously implanted with MDA PCa 183. Surgical castration was performed in 13 of these mice when tumors reached a volume of 500 mm^3^ or higher (castrated mice). Tumor volume was monitored in intact and castrated mice. Tumors from intact mice were harvested when the volume reached 2000 mm^3^ (Control). Tumors from 6 of the castrated mice were harvested at 10 days of castration (early response to castration [ERC]). The remaining seven continued to be monitored for tumor growth over time and the relapsed tumors were harvested. Relapsed tumors are those that continue to grow for two consecutive weeks after castration (Relapse). PSA was measured using PSA ELISA kit (American Qualex; San Clemente, CA, USA).

### Transcriptome analysis of CRPC MDA PCa PDXs

Samples were prepared as previously described [11]. RNA was extracted from fresh frozen tissue of 37 CRPC MDA PCa PDXs, previously characterized [16, 18] at the Biospecimen Extraction Facility (MD Anderson), using the QIAGEN RNeasy kit (Hilden, Germany). Stranded total RNA-sequencing and transcriptome analysis were performed as previously described [48], at the Advanced Technology Genomics Core (MD Anderson), using the NovaSeq 6000 SP flow cell with 100nt PE sequencing format. AR and AR downstream targets gene expression was analyzed using the pheatmap package in R [49].

### Non-targeted global metabolite profiling

Sample preparation and analysis was carried out at Metabolon, Inc (https://www.metabolon.com). In brief, sample preparation involved protein precipitation and removal with methanol, shaking and centrifugation. The resulting extracts were divided into fractions for analysis on three independent platforms: ultra-high-performance liquid chromatography/tandem mass spectrometry (UHPLC/MS/MS) optimized for basic species, UHPLC/MS/MS optimized for acidic species, and GC/MS. The details of this platform have been described previously [50]. Metabolites were identified by automated comparison of the ion features in the experimental samples to a reference library of chemical standard entries that included retention time, molecular weight (*m/z*), preferred adducts, and in-source fragments as well as associated MS spectra, and were curated by visual inspection for quality control using software developed at Metabolon [51]. Values obtained were normalized in terms of raw area counts (OrigScale). Each biochemical in OrigScale was rescaled to set the median equal to one and expressed as imputed normalized counts for each biochemical (ScaledImpData). Pathway analysis was performed by Metabolon based on information of literature and public data bases (Human Metabolome Data Base and KEGG). For statistical significance of the differentially enriched metabolites, we used the P-values derived from Welch’s two-sample *t*-test, provided by Metabolon (https://www.metabolon.com). The *P* values were corrected for multiple comparisons (*q*-value), as described by Storey J. and Tibshirani R. (2003) [52]. The q-values are provided in the supplementary material (Table S1).

### Immunohistochemistry

We performed immunohistochemistry (IHC) analyses of AR and ERG expression in mouse tissue specimens obtained from MDA PCa 183 tumors in Control (*n* = 5), ERC (*n* = 6) and Relapse (*n* = 3) groups. We also performed IHC analyses of ACAT1, OXCT1, BDH1 in sections derived from these mouse specimens and in bone biopsies obtained from the human donor of MDA PCa 183 untreated and after relapse. Donor samples were obtained from the Prostate Tissue Bank, Department of Pathology, MD Anderson Cancer Center, Houston, TX, under an Institutional Review Board approved protocol. Bone specimens were decalcified and all samples formalin-fixed, paraffin-embedded as previously described [53]. Tissue sections were stained with anti-AR (N-terminal) antibody (#M3562 Clone AR441 1:50; Dako; Santa Clara, CA, USA), anti-ERG antibody (#CM421C Clone 9FY 1:50; Biocare Medical; Pacheco, CA, USA), anti-ACAT1 antibody (#HPA004428 1:500; Sigma; Darmstadt, Germany), anti-BDH1 antibody (#HPA030947 1:2000; Sigma), anti-OXCT1 antibody (#HPA012047 1:200; Sigma), anti-HMGCL antibody (#HPA004727 1:100; Sigma), anti-HMGCS2 antibody (#HPA027442 1:1750; Sigma) and anti-SLC16A7 antibody (#HPA005911 1:100; Sigma) as described elsewhere [54].

For semi-quantitative/quantitative analysis, the stained slides were digitalized in an Aperio AT2 scanner (Leica Biosystems; Buffalo Grove, IL, USA) under 40 × objective magnification and evaluated by a pathologist (X.T.). For nuclear staining, the digital image analysis software HALO (v3.1.1076.449) (Indica Labs; Albuquerque, NM, USA) was used, and analysis was performed using a pathologist-trained specific algorithm. For cytoplasmic staining, the digital image analysis software Aperio ImageScope 12.4.3 (Leica Biosystems) was used, and analysis performed using the Positive Pixel Count algorithm 2004-08-11. In all cases, the images were annotated excluding necrotic areas and at least five areas were selected for quantification.

### Animals

All practices involving laboratory animals were conducted under the approval of the Institutional Animal Care and Use Committee of The University of Texas MD Anderson Cancer Center, under the regulation of the Animal Welfare Committee (IACUC), and conform to the NIH Policy on Humane Care and Use of Laboratory Animals.

### Statistics

Welch’s two sample *t*-test was used to ascertain statistical significance. Heatmap was created in R with the pheatmap package [49] using the Log_2_ of the ScaledImpData. Volcano plots and scatter plots were performed using EnhancedVolcano [55] and ggplots2 [56] packages in R, respectively. |Log_2_ fold change| > 1, proportion of significantly changed metabolites >0.8 and *P* value <0.05 were set as threshold for statistical interpretation [57]. For IHC quantifications, one-way ANOVA followed by Tukey’s multiple comparisons test or two-tailed *t*-test was used to assess statistical significance (*P* < 0.05), based on the number of groups being compared.

### Bioinformatics analysis

#### Information source and eligibility criteria (The Cancer Genome Atlas (TCGA) [58])

To study the impact of the expression of the selected genes on the progression-free survival (PFS) of PCa patients, we used the Xena platform [59] to access the dataset from the Prostate Adenocarcinoma Project of The Cancer Genome Atlas (TCGA-PRAD) (http://cancergenome.nih.gov/). TCGA-PRAD has gene expression data from 497 prostate tumor samples and normal adjacent tissue (last access: June 2020), measured by massively parallel sequencing (llumina HiSeq). PFS is defined as the period from the date of diagnosis until the date of the first occurrence of a new tumor event, which includes progression of the disease, locoregional recurrence, distant metastasis, new primary tumor, or death with tumor [60].

#### Information source and eligibility criteria (GEO: Gene Expression Omnibus)

To study the impact of gene expression levels on relapse-free survival (RFS) in PCa patients, we selected the Ross-Adams 2015 dataset (GSE70770) [61], according to the following criteria: [1] the study included gene expression and clinical data for each patient with ≥5 years of follow-up; [2] the study consisted of ≥60 samples; and [3] the study was published and available in public repositories. Tumor sample expression of 31,000 transcripts was measured by 47,000 probes using Illumina HumanHT-12 V4.0 expression BeadChip. This PCa patient cohort included 204 samples from men who had undergone radical prostatectomy and clinical follow-up up to eight years, including relapse information. Biochemical relapse was defined according to the European Guidelines as a prostate specific antigen (PSA) persistent rise above 0.2 ng/mL.

To study gene expression in metastatic samples, we used the following datasets: [1] GSE32269, which contains transcriptomic data from 22 primary PCa (hormone-dependent) and 29 metastatic PCa (CRPC) samples, obtained by Affymetrix Human Genome U133A Array; [2] GSE74685 dataset from Fred Hutchinson Cancer Research Center, which comprises a PCa patient’s cohort with samples from primary (*n* = 14) or metastatic (*n* = 149) tumors from 63 PCa patients (20 bone metastasis, 21 liver metastasis, 69 lymph node metastasis, 22 lung metastasis and 17 other metastasis), with complete Agilent 44 K whole human genome expression oligonucleotide microarray.

#### Information source (SU2C/PCF Dream Team 2019 data set (SU2C)) [62]

We used the data set from the SU2C-PCF Dream Team: Precision Therapy for Advanced Prostate Cancer that has whole-exome sequencing of 444 castrate-resistant prostate cancer tumor. It contains clinical, transcriptomic and survival information for 71 metastatic samples from CRPC tumors. Data was accessed using the cBioPortal web tool (https://www.cbioportal.org/).

For *AR/ERG* gene expression categorization in patients that biochemically relapsed within five years from the Ross–Adams dataset (GSE70770), we established the median of each gene expression level as the cutoff point (>median: high expression; <median: low expression). Two tailed *t*-test was used to assess the statistical significance using GraphPad Prism software (La Jolla, CA, USA). Survminer R package [63] was used to explore patients’ RFS and PFS, and to generate Kaplan–Meier (KM) curves. For time to event analysis, we used the minimal *P* value approach from the Cutoff Finder tool [64] to categorize patients into two groups based on the gene expression levels. For univariable and multivariable analyses, the log-rank test and Cox proportional hazard model regression were employed. Statistical significance was set as *P* < 0.05.

## Supplementary information

Supplementary Figures

Supplementary Table 1
