# Supplementary material for: Prostate cancer castrate resistant progression usage of non-canonical androgen receptor signaling and ketone body fuel
Source: Oncogene. 2021 Sep 28;40(44):6284–98. doi: 10.1038/s41388-021-02008-9 (PMC8566229; doi:10.1038/s41388-021-02008-9)
Supplement: Supplementary file 1 — Supplementary Figures [file 41388_2021_2008_MOESM1_ESM.docx]

SUPPORTING INFORMATION

**
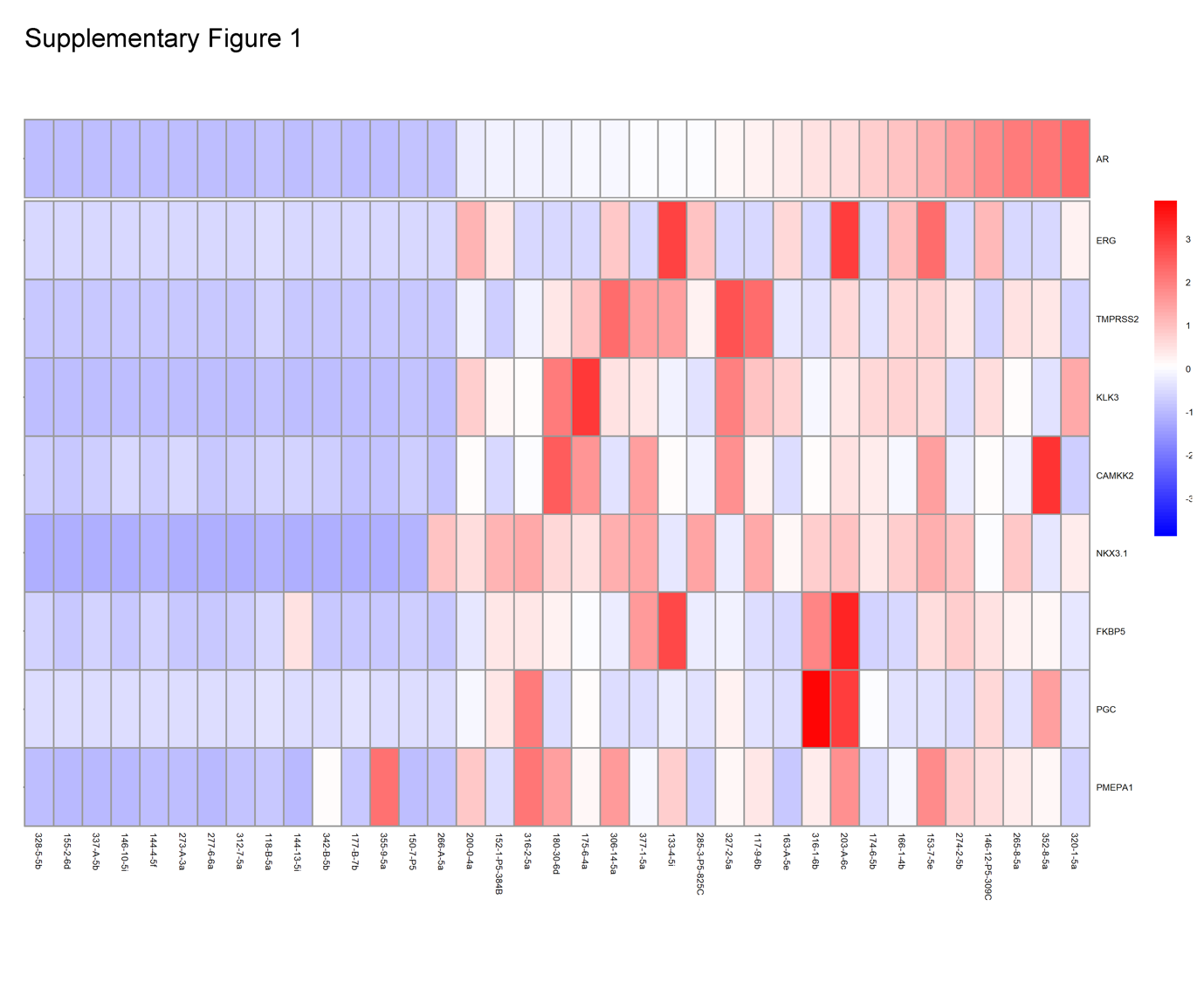
Fig S1. *AR* and AR downstream targets’ gene expression in CRPC MDA PCa PDXs.** Analysis of RNAseq data from 37 CRPC MDA PCa PDXs showcased in a heatmap depicting *AR* and AR downstream targets’ (*ERG*, *TMPRSS2*, *KLK3*, *CAMKK2*, *NKX3.1*, *FKBP5*, *PGC*, *PMEPA1*) gene expression. CRPC PDXs identifiers are shown in the x-axis. Red, white, and blue represent greater, intermediate, and lower gene expression levels, respectively. Expression values are expressed as z-score.


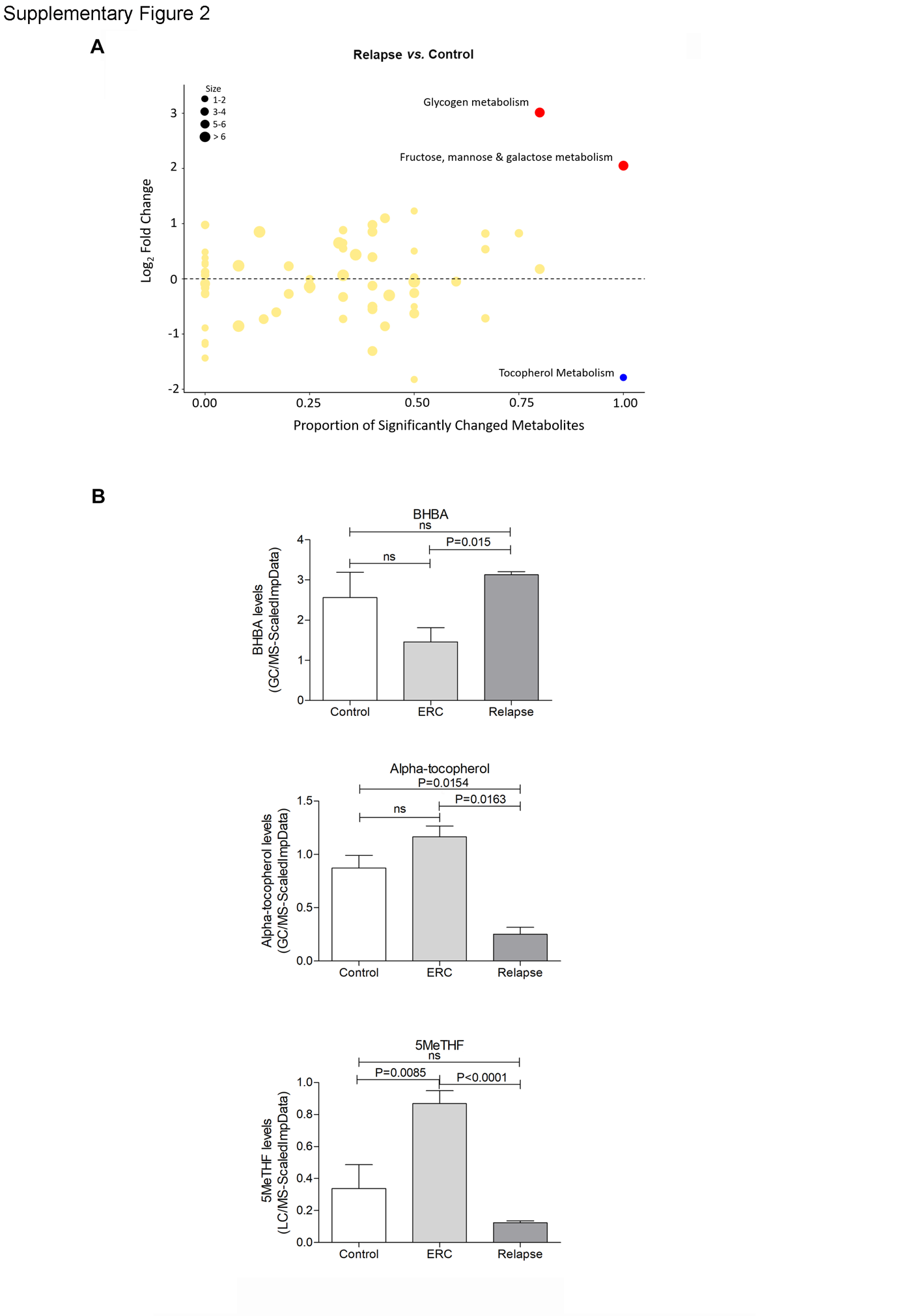


**Fig S2. Metabolic shift of MDA PCa 183 Relapse *vs*. Control and relative abundance of metabolites altered in Relapse *vs.* ERC. (A)** Scatter plots depicting altered KEGG pathways according to the metabolites increased and decreased in Relapse *vs*. Control using Welch's two-sample *t*-test. The x-axis indicates the proportion of metabolites in a pathway that are significantly changed (both increased and decreased) in the comparison. The y-axis plots the average Log_2_ Fold Change of these metabolites. A proportion of significantly changed metabolites of 0.8 and a |Log_2_ Fold Change| > 1 were set as thresholds for pathways enrichment. (B) Bar plots of the abundance of BHBA (3-hydroxybutyrate), alpha-tocopherol and 5MeTHF (5-methyltetrahydrofolate) measured by GC/MS or LC/MS in Control (n=7), ERC (n=6) and Relapse (n=3) mice. Statistical significance was assessed using Welch's two-sample *t*-test between each comparison and was set at P<0.05. Values represent the ScaleImpData for each metabolite. Error bars: SEM.

**
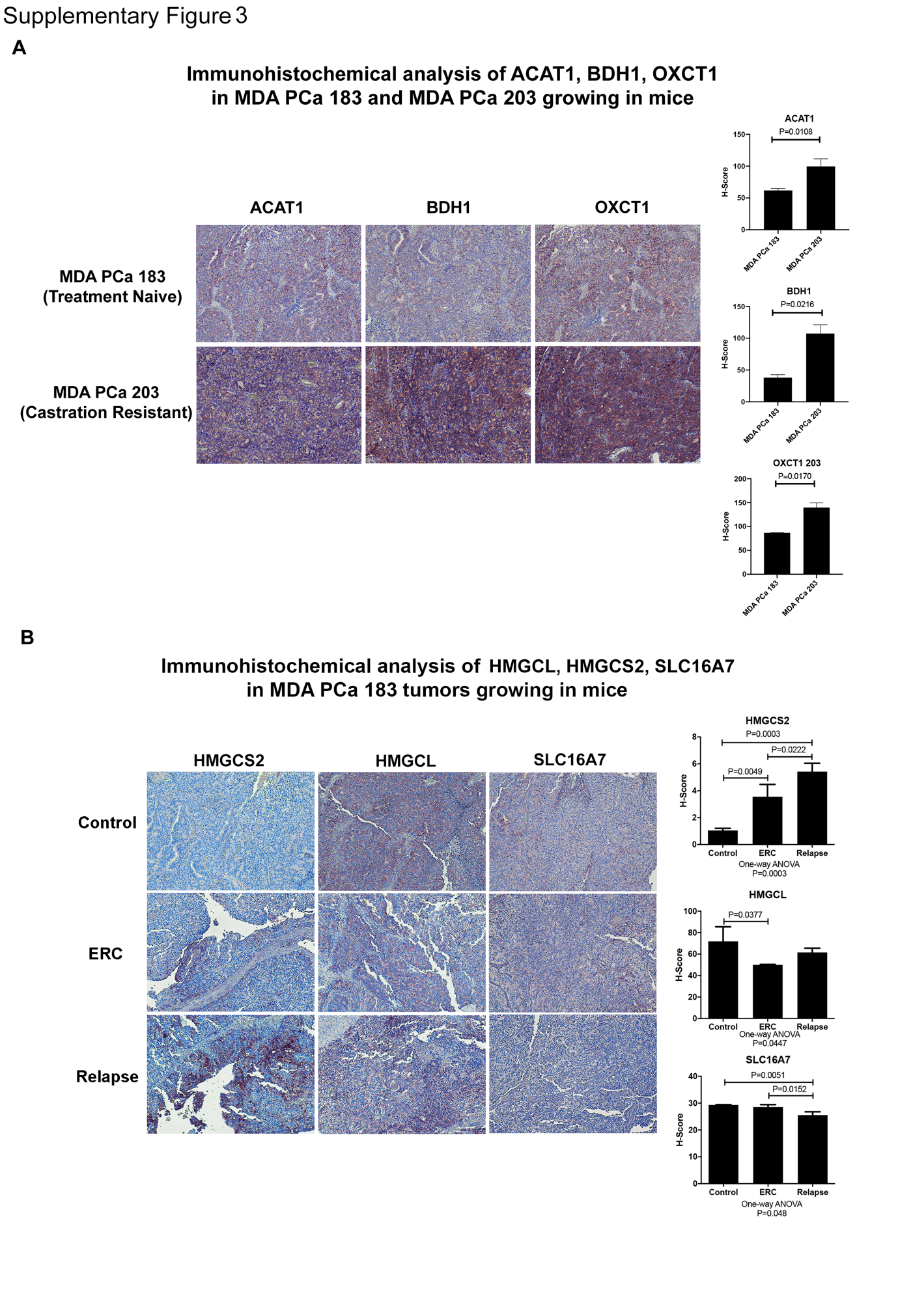
**

**Fig S3. Expression of enzymes and transporters involved in ketone body metabolism in preclinical progression of PCa.** (A) Representative photomicrograph images of sections of samples of MDA PCa 183 and MDA PCa 203 longitudinal samples from same human donor before ADT (treatment naive) and after progression (castration resistant), respectively, and corresponding IHC quantification. Magnification 100X. Data are represented as mean ± SD. Unpaired two-tailed *t*-test was used to asses statistical significance (P<0.05). Samples were immunostained for ACAT1, OXCT1 and BDH1. (B) Representative photomicrograph images of sections of MDA PCa 183 tumors growing in mice and corresponding IHC quantification (Control [n=5], ERC [n=6] and Relapse [n=3]). Magnification 100X. Data are represented as mean ± SD. One-way ANOVA followed by Tukey’s multiple comparisons test was used to asses statistical significance (P<0.05). Samples were immunostained for HMGCL, HMGCS2 and SLC16A7. ERC: early response to castration; ACAT1: acetyl-CoA acetyltransferase; OXCT1: 3-oxoacid CoA-transferase 1; BDH1: 3-Hydroxybutyrate Dehydrogenase 1; HMGCS2: hydroxy-methylglutaryl-CoA synthase 2; HMGCL: hydroxy-methylglutaryl-CoA lyase; SLC16A7: monocarboxylate transporter 2.


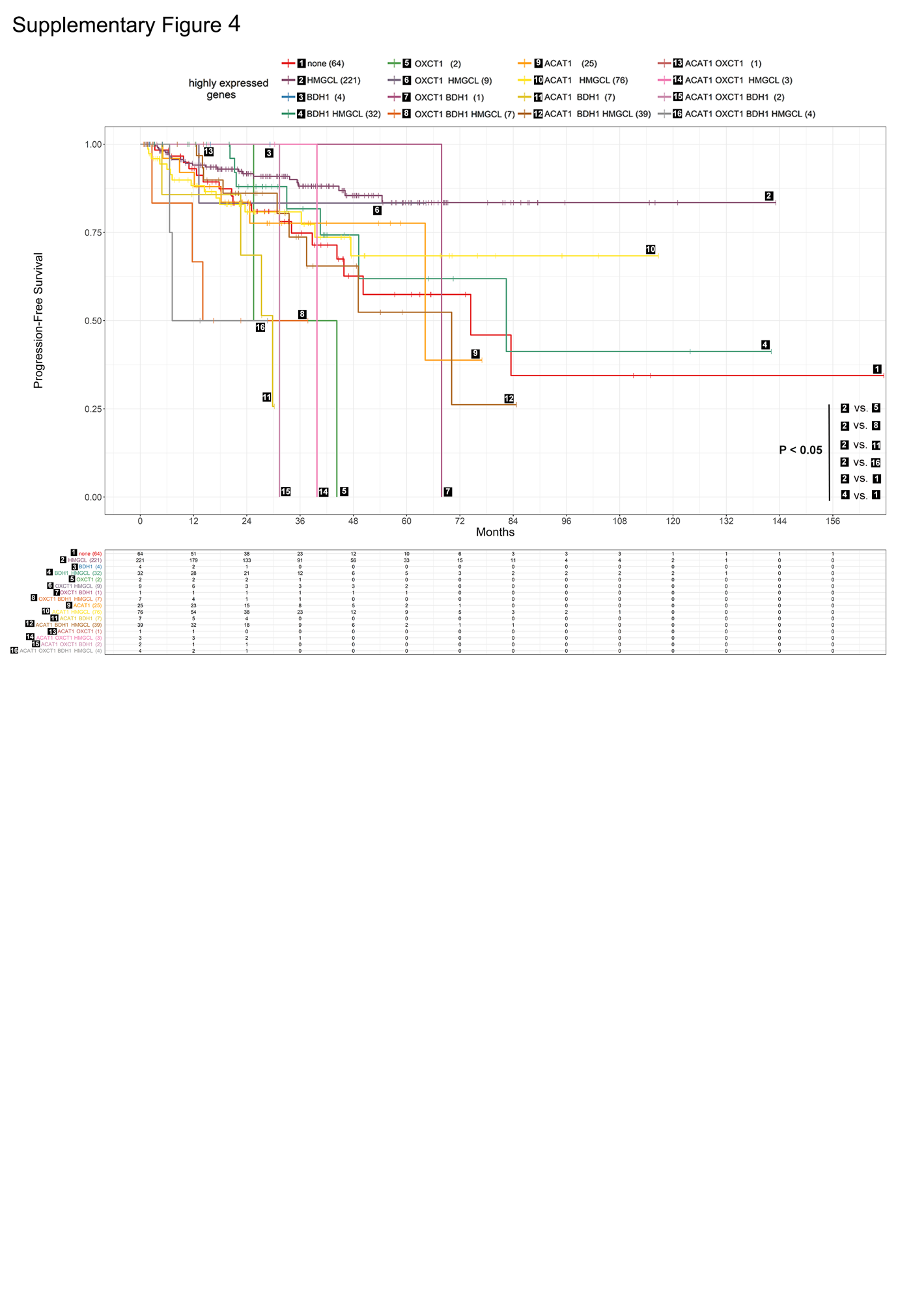


**Fig S4. Progression free survival in TCGA-PRAD subgroups.** KM curves for PFS in months for PCa patients subgroups with different expression levels of *ACAT1*, *OXCT1*, *BDH1* and *HMGCL* in TCGA-PRAD: (1) low *ACAT1*, *OXCT1, BDH1* and *HMGCL* expression (n=64); (2) low *ACAT1*, low *OXCT1, low BDH1* and high *HGMCL* expression (n=221); (3) high *BDH1* and *HMGCL* and low *ACAT1* and *OXCT1* expression (n=32), (4) low *ACAT1* and high *OXCT1*, *BDH1* and *HMGCL* expression (n=7), (5) high *ACAT1* and *BDH1* and low *OXCT1* and *HMGCL* expression (n=7); (6) high *ACAT1, OXCT1*, *BDH1* and *HMGCL* expression (n=4). The table indicates the number of patients assessed every 12 months. Log-rank test was employed to assess statistical significance. All comparisons considered low expression patients as the reference group. HR: hazard ratio. Statistical significance: P<0.05.

**
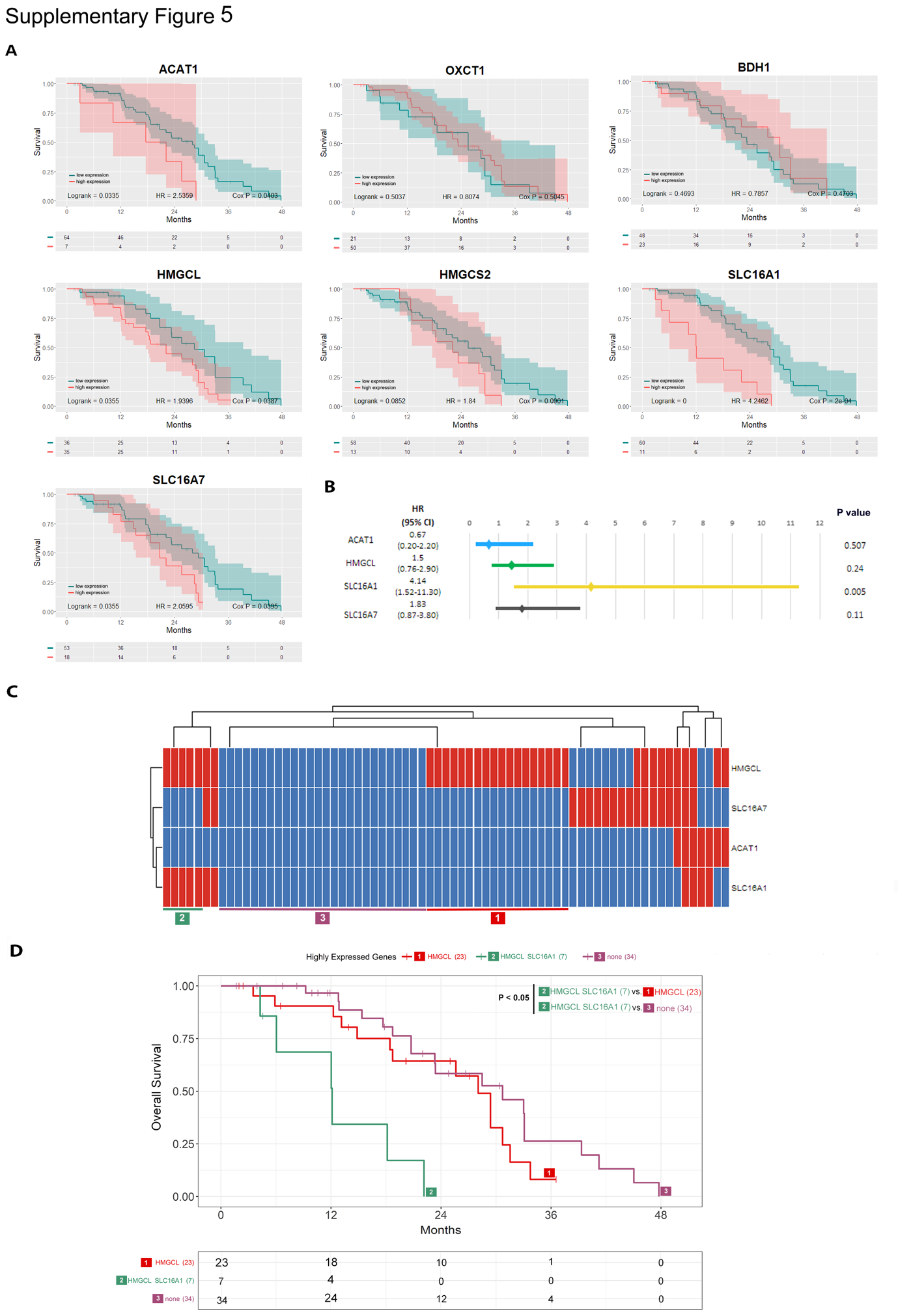
**

**Fig S5. Clinical outcome of ketogenic/ketolytic enzymes and MCT transporters in CRPC metastatic samples from the SU2C dataset.** (A) Kaplan–Meier (KM) curves for overall survival (OS) in months for PCa patients with low (green) or high (red) *ACAT1, OXCT1, BDH1, HMGCL, HMGCS2, SLC16A1* and *SLC16A7* expressions in the SU2C dataset metastatic samples (n=71). Log-rank test and Cox proportional hazard model regression were employed to assess statistical significance. (B) Multivariable Cox proportional hazard model regression analysis for *ACAT1*, *HMGCL*, *SLC16A1* and *SLC16A7* presented as forest plots for OS. (C) Heatmap depicting low (blue) or high (red) *ACAT1*, *HMGCL, SLC16A1*, and *SLC16A7* mRNA expression for PCa patients according to the SU2C dataset. (D) KM curves for OS in months for metastatic PCa patients subgroups with different expression levels of *ACAT1*, *HMGCL, SLC16A1* or *SLC16A7* in SU2C: (1) low *ACAT1*, *SLC16A1, SLC16A7* and high *HMGCL* expression (n=23); (2) low *ACAT1* and *SLC16A7* and high *HMGCL* and *SLC16A1* expression (n=7); (3) low expression for *ACAT1*, *HMGCL*, *SLC16A1* and *SLC16A7* (n=34). The table indicates the number of patients assessed every 12 months. Log-rank test was employed to assess statistical significance. All comparisons considered low expression patients as the reference group. HR: hazard ratio. Statistical significance: P<0.05. All comparisons considered low expression patients as the reference group. HR: hazard ratio. Statistical significance: P<0.05.


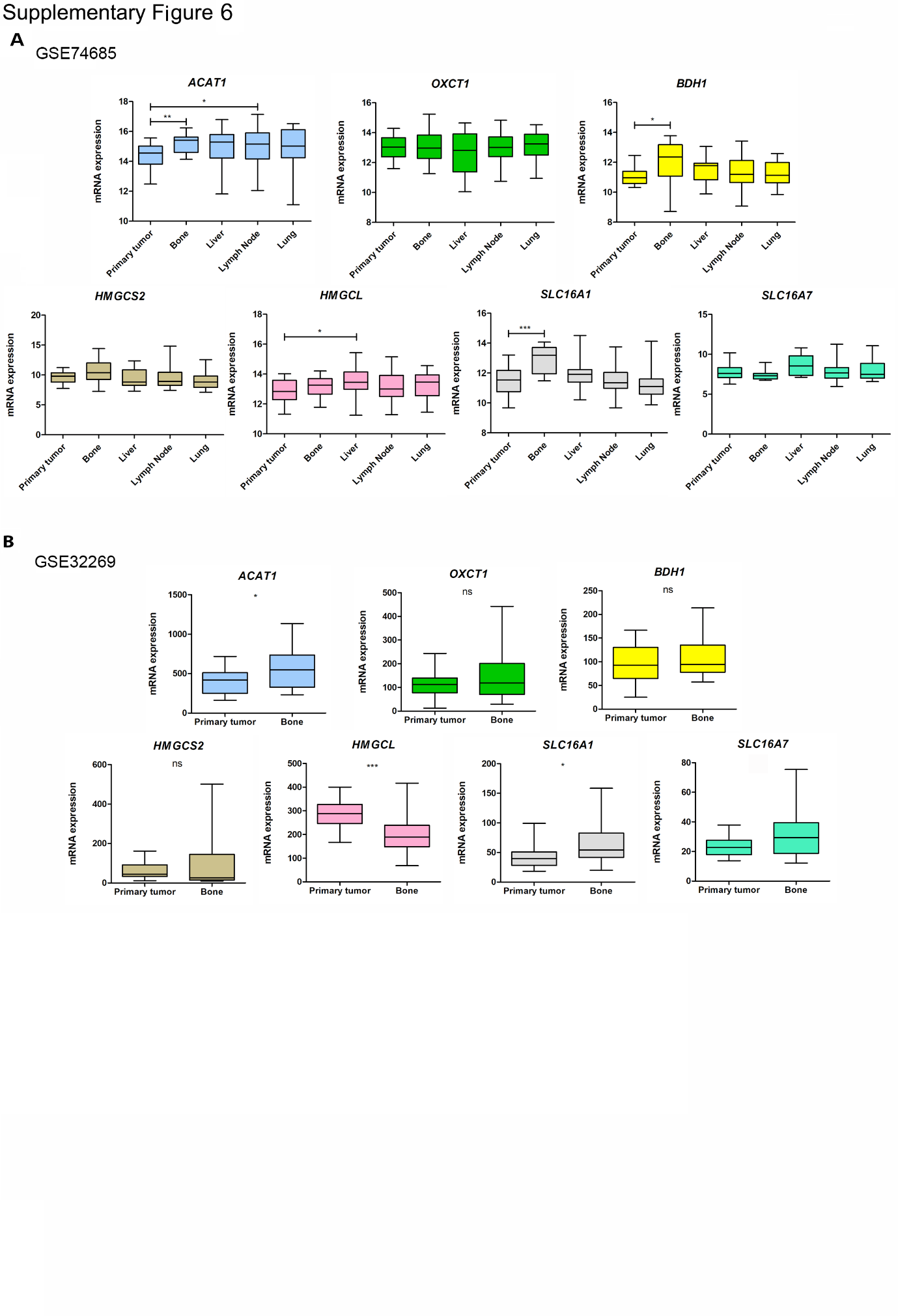


**Fig S6.** **Bioinformatics** **analysis of ketogenic/ketolytic enzymes and MCT transporters in PCa metastatic samples.** (A) Gene expression analysis for *ACAT1*, *OXCT1*, *BDH1*, *AR,* *ERG, HMGCS2, HMGCL, SLC16A1* and *SLC16A7* comparing primary tumors (n=14) *vs*. different metastatic samples (20 bone metastasis, 21 liver metastasis, 69 lymph node metastasis, and 22 lung metastasis) (GSE74685; n=149). (B) Gene expression analysis for *ACAT1*, *OXCT1*, *BDH1*, *AR,* *ERG, HMGCS2, HMGCL, SLC16A1* and *SLC16A7* comparing primary tumors (n=22) *vs*. CRPC bone metastasis (29) (GSE32269; n=51). Data are presented as box-and-whisker plots (min-max error bars). Mann-Whitney U test was used to assess statistical significance between primary tumors and metastatic sites since normal distribution was not observed for all groups. Statistical significance: P<0.05. *P<0.05; **P<0.001; ***P<0.0001.
